# Elevated plasma endocan and BOC in heart failure patients decrease after heart transplantation in association with improved hemodynamics

**DOI:** 10.1007/s00380-020-01656-3

**Published:** 2020-07-10

**Authors:** Salaheldin Ahmed, Abdulla Ahmed, Habib Bouzina, Jakob Lundgren, Göran Rådegran

**Affiliations:** 1 Department of Clinical Sciences Lund, Cardiology, Lund University, Lund, Sweden; 2 The Hemodynamic Lab, The Section for Heart Failure and Valvular Disease, VO. Heart and Lung Medicine, Skåne University Hospital, Lund, Sweden

**Keywords:** Biomarkers, Heart failure, Heart transplantation, Hemodynamics, Pulmonary hypertension

## Abstract

**Background:**

The prevalence of heart failure (HF) is rising with ageing population and constitutes a major health problem globally. A common complication of HF is pulmonary hypertension (PH) which negatively impacts survival. A pathophysiological association between HF and PH with tumorigenic processes has been suggested. We aimed to identify the plasma levels of, and the association between tumour-related proteins and hemodynamic improvements in patients with HF and PH due to left heart disease (LHD) before and 1-year after heart transplantation (HT).

**Methods:**

Forty-eight tumour-related proteins were measured with proximity extension assay in plasma from 20 controls and 26 HF patients before and 1-year after HT. Patients’ hemodynamics were measured with right heart catheterization.

**Results:**

Out of 48 proteins, specifically, plasma levels of endocan and brother of CDO (BOC) were elevated in end-stage HF patients compared to controls (*p* < 0.001), but decreased after HT (*p* < 0.01), towards controls’ levels. The decrease of endocan levels after HT correlated with improved mean pulmonary arterial pressure (*r*_s_ = 0.80, *p* < 0.0001), pulmonary arterial wedge pressure (*r*_s_ = 0.63, *p* = 0.0012), and pulmonary vascular resistance (*r*_s_ = 0.70, *p* < 0.001). The decrease and normalization of BOC after HT correlated with decreased mean right atrial pressure (*r*_s_ = 0.61 *p* = 0.0015) and NT-proBNP (*r*_s_ = 0.57, *p* = 0.0022), as well as increased cardiac index (*r*_s_ = − 0.51, *p* = 0.0086) and left-ventricular stroke work index (*r*_s_ = − 0.57, *p* = 0.0039).

**Conclusion:**

Our results suggest that (i) plasma endocan in HF may reflect the state of pulmonary vascular congestion and PH-LHD, whereas (ii) plasma BOC may reflect the cardiac function and the hemodynamic overload in HF. The exact role of these proteins and their clinical applicability as biomarkers in HF and PH-LHD ought to be investigated in larger cohorts.

**Electronic supplementary material:**

The online version of this article (10.1007/s00380-020-01656-3) contains supplementary material, which is available to authorized users.

## Introduction

Heart failure (HF) is a clinical syndrome with a prevalence of 1–2% of the adult population in developed countries [1]. Despite improved treatment modalities in the last 2 decades, the 5-year survival of HF patients with reduced ejection fraction remains poor [2]. A common complication in HF, irrespective of ejection fraction, is pulmonary hypertension (PH), with negative impact on survival and exercise capacity [3]. PH may arise as a consequence of left heart disease (LHD), through congestion and backward transmission of elevated left-sided filling pressures. A sustained congestion may cause endothelial dysfunction and excessive vasoconstriction with subsequent vascular remodeling [4].

The chronic progression of HF involves an array of different pathophysiological mechanisms [5]. Proteomic biomarkers are emerging as a new tool for diagnosis and prognosis, and may reflect this pathophysiological progression [5, 6]. In the fields of HF [7] and especially PH, biomarker research is of particular interest, as outlined in a recent state-of-the-art review of PH pathology and pathobiology [8]. To date, however, the clinical use of biomarkers in HF and PH remains mainly limited to natriuretic peptides and its precursors, which collectively reflect one pathophysiological pathway [9, 10].

Although tumorigenic processes and HF are two distinct entities, recent studies reported that HF could prime the onset of cancer by mechanisms involved in pathophysiology of HF, such as aberrant neuro-hormonal axis and growth hormonal overexpression with impact on proliferation [11, 12]. Additionally, the Warburg effect, originally ascribed to cancer cells undergoing higher glycolytic activity through fermenting glucose to lactate during normoxic conditions, has also been postulated to be involved in the pathobiology of HF and pulmonary arterial hypertension (PAH). For instance, in hypertrophic cardiomyopathy, metabolic dysfunction and energy deficit are functional in cardiac dysfunction and ventricular remodeling [13]. Moreover, the development of pulmonary vascular remodeling during PAH progression, which pathobiologically may imitate that observed in PH-LHD [14], involves cellular acquisition of tumorigenic traits including deranged cellular energetics, sustained proliferative signaling, and reduced susceptibility to apoptosis [15].

Intriguingly, several studies have appraised tumour-related proteins in the context of either PAH [16, 17] or HF [18]. In a murine model, deletion of the pro-apoptotic transcription factor P53 exacerbated hypoxia-induced PAH [16]. A subsequent study showed that treatment with Nutilin-3a, a *cis*-imidazoline analog that stabilizes the pro-apoptotic transcription factor p53 and increases the pro-senescent p21 expression, reversed PAH in mice and induced cell growth arrest and senescence in cultured human pulmonary arterial smooth muscle cells [17]. Mucin-16 or CA125, a marker of ovarian cancer, is, furthermore, elevated in HF patients with severe fluid overload and may be of prognostic value [18]. To our knowledge, there is, however, a paucity in studies with focus on tumour markers in HF and PH-LHD as well as the consequence of heart transplantation (HT).

In search of potential biomarkers reflecting alterative pathophysiological pathways in HF and related PH, such as inflammatory response, cellular proliferation, and endothelial dysfunction, we aimed to identify the levels of tumour-related proteins with associated hemodynamic improvements in HF and PH-LHD, before and after HT. Identifying such proteins may aid in generating hypothesis for clinical research and the incorporation of a multi-marker testing panel of different pathophysiological mechanisms. Moreover, a biomarker-guided phenotyping of HF and related PH may potentially optimize the clinical management and prompt the development of new therapies [6], especially in the stagnant supply of donor hearts enabling HT [19].

## Materials and methods

### Study population

The present study was based on 29 patients with end-stage HF with or without preoperative PH evaluated before and 1-year after HT at Skåne’s University Hospital in Lund, Sweden, as well as 20 cardiopulmonary healthy controls (≥ 18 years) with no history of ischemic heart disease, atrial fibrillation, stroke, or diabetes mellitus. Although two of the controls reported a previous thyroid illness, all were included as none of the controls exhibited cardiovascular-related comorbidities. Patients with PH after HT (*n* = 1) and with missing postoperative hemodynamic data (*n* = 2) were excluded. Left-ventricular dysfunction was diagnosed according to the routine clinical investigation with echocardiography and/or magnetic resonance imaging [1]. Informed written consent was acquired from all participants. The population has previously been characterized, including patients’ plasma creatinine and NT-proBNP, shown in Table 1 [20, 21]. Briefly, 50% of the controls were male, had a median age of 41 years and a median body surface area (BSA) of (1.92 m^2^, *n* = 19).

**Table 1:** Demographic data of heart failure patients following heart transplantation

Variable	Pre-HT (*n* = 26)		Post-HT (*n* = 26)	
	*n*	Median (IQR)	*n*	Median (IQR)
Female, *n* (%)	5 (19.2)			
Age (years)	26	50 (45–61)*	26	52 (47–63)
Height (cm)	26	178 (172–180)	26	177 (172–181)
Weight (kg)	25	80 (71–89)	26	78 (69–90)
BSA (m^2^)	25	2 (1.8–2.1)	26	2 (1.8–2.1)
Creatinine (μmol/L)	25	108 (90–123)	26	114 (97–142)^§^
eGFR (mL/min/1.73 m^2^)	25	63 (55–71)	26	53 (43–72)^§^
Atrial fibrillation, *n* (%)	26	13 (50)	26	–
Hypertension, *n* (%)	26	5 (19.2)	26	3 (11.5)
Diabetes mellitus, *n* (%)	26	3 (11.5)	26	9 (34.6)
HF and PH classification	*n* (%)		*n* (%)	
HFrEF (EF < 50%)	24 (92.3)		–	
HFpEF (EF ≥ 50%)	2 (7.7)		–	
PH	19 (73.1)^#^		–	
Ipc-PH	10 (38.5)		–	
Cpc-PH	9 (34.6)		–	
HF aetiology			–	
DCM	17 (65.4)		–	
HCM	3 (11.5)		–	
ICM	3 (11.5)		–	
Other	3 (11.5)		–	
Medications				
β-Blockers	25 (96.2)		9 (34.6)	
ACEi	11 (42.3)		–	
ARB	11 (42.3)		10 (38.5)	
MRA	22 (84.6)		3 (11.5)	
Furosemide	24 (92.3)		12 (46.2)	
Cordarone	4 (15.4)		–	
Prednisolone	1 (3.8)		25 (96.2)	
Cyclosporine	–		3 (11.5)	
Tacrolimus	–		23 (88.5)	
Mycophenolate mofetil	–		21 (80.8)	
Azathioprine	–		5 (19.2)	

*HT* heart transplantation, *IQR* interquartile range, *BSA* body surface area = (weight^0.425^ × height^0.725^) × 0.007184 [14], *PH* pulmonary hypertension, *HFrEF* heart failure with reduced ejection fraction, *HFpEF* heart failure with preserved ejection fraction, *Ipc-PH* isolated post-capillary PH, *Cpc-PH* combined post-capillary and pre-capillary PH, *DCM* dilated cardiomyopathy, *HCM* hypertrophic CM, *ICM* ischemic CM, *ACEi* angiotensin-converting enzyme inhibitor, *ARB* angiotensin II receptor blocker, *MRA* mineralocorticoid receptor antagonist. Notably, 84.6% of the patients had either ACEi or ARB. These data have previously been published [20]

*p < 0.0001, FDR (Q = 0.01); vs. control

^§^Nonsignificant vs. pre-HT

^#^One patient suffered from severe orthopnea, hence the unsuccessful PAWP assessment. After optimization with furosemide and levosimendan, subsequent right heart catheterization confirmed Ipc-PH. Estimated glomerular filtration rate was calculated with the revised Lund–Malmö formula [62]

The study was conducted in accordance with the declarations of Helsinki and Istanbul and approved by the ethical board in Lund, Sweden (diary numbers: 2010/114; 2010/442; 2011/368; 2011/777; 2014/92 and 2015/270).

### Protein analysis

Venous blood samples were collected from the venous introducer of the patients’ internal jugular veins during right heart catheterization (RHC) and from peripheral veins in controls, stored at − 80 °C in Lund Cardio Pulmonary Register (LCPR), a cohort of Region Skåne’s biobank. As per protocol, neither the patients nor controls were fasting during blood sample collection. Plasma aliquots, retrieved from LCPR, were analysed with proximity extension assay (PEA). PEA is based on the use of oligonucleotide-linked antibodies and qPCR for protein detection and quantification (Proseek Multiplex Cardiovascular II, III and Oncology II kits, Olink, Proteomics, Uppsala, Sweden) [22]. Proteins analysed were N-terminal pro b-type natriuretic peptide (NT-proBNP), 5′-nucleotidase, protein AMBP (AMBP), aminopeptidase N (AP-N), bleomycin hydrolase (BLM-H), brother of cell adhesion molecule-related/down-regulated by oncogenes (CDO) or (BOC), carbonic anhydrase 9 (CA9), cathepsin Z, p21 or cyclin-dependent kinase inhibitor 1A (CDKN1A), carcinoembryonic antigen-related cell adhesion molecule (CEACAM) 1 and 5, contactin-1, cornulin, carboxypeptidase A1 (CPA1), carboxypeptidase B (CPB1), carboxypeptidase E (CPE), cystatin B, endothelial cell-specific molecule 1 or endocan, epithelial cell adhesion molecule (Ep-CAM), furin, gastrotropin, glyoxalase I or lactoylglutathione lyase, kallikrein 6, 8, 11, 13 and 14, Ly6/PLAUR domain-containing protein 3 (LYPD3) or C4.4A, mesothelin, methionine aminopeptidase 2 (MetAP2), melanoma-derived growth regulatory protein or melanoma inhibitory activity (MIA), midkine, mucin-16 or CA125, podocalyxin, prostasin, PVRL4 or nectin-4, S100A11, S100A4, secretory carrier-associated membrane protein 3 (SCAMP3), secretoglobin family 3A member 2 (SCGB3A2), tyrosine-protein phosphatase non-receptor-type substrate 1 (SHPS-1), sortilin, T-cell leukemia/lymphoma protein 1A (TCL1A), trefoil factor 3 (TFF3), protein-glutamine gamma-glutamyltransferase 2 (TGM2), WAP four-disulfide core domain protein 2 (WFDC2), vimentin, V-set and immunoglobulin domain-containing protein 2 (VSIG2), and Xaa-Pro aminopeptidase 2 (XPNPEP2). NT-proBNP and all 48 proteins are expressed arbitrarily in linear normalized protein expression scale. PEA’s analytical quality in assessing proteins is rigorously validated regarding sensitivity, dynamic range, specificity, precision, and scalability. Panel and protein-specific validation documents can be found on www.olink.com/downloads.

### Right heart catheterization

As a part of the clinical evaluation for HT, the patients’ hemodynamic profiles were characterized by cardiologists before and during the routine 1-year follow-up after HT by RHC, using a Swan-Ganz catheter (Baxter Health Care Corp, Santa Ana, CA, USA) inserted through the right internal jugular vein. Recorded parameters were systolic pulmonary arterial pressure (sPAP), diastolic PAP (dPAP), mean PAP (mPAP), mean right atrial pressure (MRAP), pulmonary arterial wedge pressure (PAWP), mixed venous oxygen saturation (SvO_2_), and arterial oxygen blood saturation (SaO_2_). Mean arterial pressure (MAP) was measured non-invasively and thermodilution was used to estimate cardiac output (CO).

### Hemodynamic definitions

The other hemodynamic parameters were calculated with the following formulas: cardiac index (CI) = CO/BSA, stroke volume (SV) = CO/heart rate, stroke volume index (SVI) = SV/BSA, transpulmonary pressure gradient, (TPG) = mPAP − PAWP, pulmonary vascular resistance (PVR) = TPG/CO, PVR index (PVRI) = TPG/CI, diastolic pulmonary pressure gradient (DPG) = DPAP − PAWP, right-ventricular stroke work index, (RVSWI) = (mPAP − MRAP) × SVI, left-ventricular stroke work index (LVSWI) = (MAP − PAWP) × SVI, pulmonary arterial compliance, (PAC) = SV/(sPAP − dPAP), and arteriovenous oxygen difference (a − vO_2_diff) = (SaO_2_ − SvO_2_) × plasma hemoglobin × 1.34.

PH-LHD was defined by a resting mPAP ≥ 25 mmHg, PAWP > 15 mmHg and sub-classified into isolated post-capillary PH (DPG < 7 mmHg and/or PVR ≤ 3 WU) or combined post-capillary and pre-capillary PH (DPG ≥ 7 and/or PVR > 3 WU), according to current guidelines [10]. HT were performed at Skåne’s University Hospital in Lund, Sweden, according to the International Society for Heart and Lung Transplantation guidelines [23, 24].

### Hemodynamic improvement

Hemodynamic data of patients before and 1-year after HT have previously been described [20, 21], with an additional subgroup description of patients with HF without PH (Table 2 and supplementary Table 1, respectively).

**Table 2:** Key hemodynamic parameters of patients before and 1-year after heart transplantation

Hemodynamic parameter	Pre-HT (*n* = 7)		Post-HT (*n* = 7)		Pre-HT (*n* = 19)		Post-HT (*n* = 19)		Pre-HT (*n* = 26)		Post-HT (*n* = 26)		*p* value
	*n*	Median (IQR)	*n*	Median (IQR)	*n*	Median (IQR)	*n*	Median (IQR)	*n*	Median (IQR)	*n*	Median (IQR)	Post-HT vs. Pre-HT
MAP (mmHg)	6	87 (74–96)	7	107 (94–116)	19	82 (78–90)	19	101 (90–106)	25	82 (77–93)	26	102 (91–108)	1.2 × 10^–6^*
mPAP (mmHg)	6	18 (17–21)	7	16 (13–18)	19	31 (29–39)	19	13 (12–17)	25	29 (24–38)	26	14 (12–17)	1.8 × 10^–7^*
PAWP (mmHg)	6	14 (11–18)	7	7 (6–10)	18	23 (19–27)	19	6 (4–8)	24	20 (18–25)	26	7 (4–9.3)	2.4 × 10^–7^*
MRAP (mmHg)	6	11 (3.8–19)	7	3 (2–6)	19	14 (9–17)	18	2.5 (0–4)	25	14 (7.5–18)	25	3 (1–4)	4.4 × 10^–6^*
CO (L/min)	6	3.6 (3–4.7)	7	5.6 (5–7.2)	19	3.2 (2.6–4)	19	5.4 (4.9–6.5)	25	3.3 (2.6–4.1)	26	5.5 (5–6.5)	6 × 10^–8^*
CI (L/min/m^2^)	6	1.9 (1.6–2.2)	7	2.7 (2.2–3.7)	19	1.6 (1.4–2.1)	19	2.9 (2.6–3.2)	25	1.8 (1.4–2.2)	26	2.8 (2.6–3.2)	1.2 × 10^–7^*
SVI (mL/beat/m^2^)	6	27 (22–29)	7	39 (30–42)	19	23 (18–29)	19	36 (34–39)	25	25 (18–29)	26	36 (33–40)	1.2 × 10^–7^*
PVR (WU)	6	1.2 (0.85–1.4)	7	1.6 (0.41–1.7)	18	3.2 (2.3–3.6)	19	1.4 (0.9–1.9)	24	2.4 (1.4–3.5)	26	1.4 (0.89–1.9)	6.5 × 10^–5^*
PAC (mL/mmHg)	6	4.6 (3.1–10)	7	6.2 (4.2–9.3)	19	1.8 (1.7–3.1)	19	5.1 (4–6.3)	25	2.2 (1.8–3.1)	26	5.4 (4.1–6.6)	0.00029*
LVSWI (mmHg × mL/m^2^)	6	1907 (1252–2406)	7	3498 (2971–3789)	17	1470 (934–1841)	19	3275 (3116–3873)	24	1541 (1052–2007)	26	3344 (3167–3810)	1.2 × 10^–7^*

*IQR* interquartile range, *WU* wood units, *MAP* mean arterial pressure, *mPAP* mean pulmonary arterial pressure, *PAWP* pulmonary arterial wedge pressure, *MRAP* mean right atrial pressure, *CO* cardiac output, *CI* cardiac index, *SVI* stroke volume index, *PVR* pulmonary vascular resistance, *PAC* pulmonary arterial compliance, *LVSWI* left-ventricular stroke work index

*p < 0.0003. FDR(Q = 0.01). Subgroup hemodynamics: (*n* = 7) heart failure patients without pulmonary hypertension and (*n* = 19) patients with pulmonary hypertension due to left heart disease. A missing CO value was calculated by indirect Fick instead of thermodilution before HT. Parts of this table have been previously published [21]

### Statistics

Continuous data are presented as median (interquartile range). Distribution assumptions of normality were determined visually, using histrograms. As the data were non-Gaussian distributed, Wilcoxon signed-rank test and Mann–Whitney *U* test were used as appropriate. Correlation analysis of changes [∆, (Post-HT) − (Pre-HT)] was expressed by Spearman’s rank correlation coefficient (*r*_s_). The two-stage step-up procedure of false discovery rate (FDR) was used to adjust for mass significance [25] and *p* values less than attained thresholds were considered statistically significant. *Q* values were set at 0.01 for *t* tests and 0.1 for correlations. Statistical analyses were performed using Prism version 8.01 for Windows, GraphPad Software, La Jolla California USA, www.graphpad.com).

### Study set-up

**Fig. 1:**
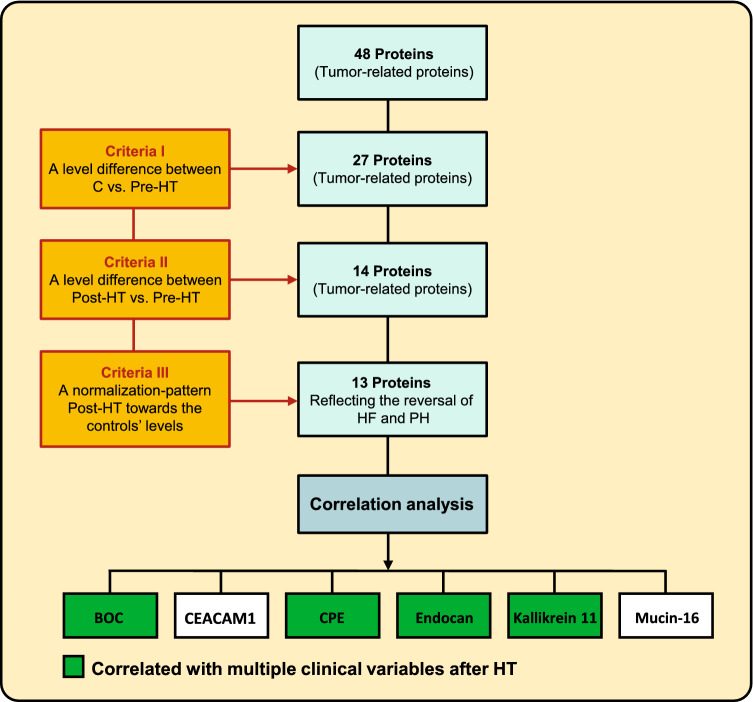
Study set-up and biomarker selection. *C* control, *HF* heart failure, *HT* heart transplantation, *PH* pulmonary hypertension, *Post-HT* 1-year after HT, *BOC* brother of CDO, *CEACAM1* carcinoembryonic antigen-related cell adhesion molecule 1, *CPE* carboxypeptidase E

To identify plasma proteins reflecting the reversal of HF in response to HT, three criteria were set; (i) a significant plasma-level difference pre-HT vs. post-HT, (ii) a significant plasma-level difference in controls vs. pre-HT, and (iii) a plasma-level change of post-HT towards controls’ levels, resembling that of NT-proBNP, (FDR, Q = 0.01). Next, proteins reflecting a pattern consistent with the reversal of HF in response to HT were correlated with NT-proBNP and improved hemodynamic parameters of heart and pulmonary circulation, i.e., mPAP, MRAP, PAWP, PVR, PAC, CI, and LVSWI (FDR , Q = 0.1). Proteins correlating to several parameters were of particular interest; and a subgroup analysis between PH-LHD and HF without PH was performed for these proteins thereafter. The study set-up is summarized in Fig. 1.

### Results

### Plasma endocan, BOC, CPE, and kallikrein 11 in end-stage heart failure patients

**Fig. 2:**
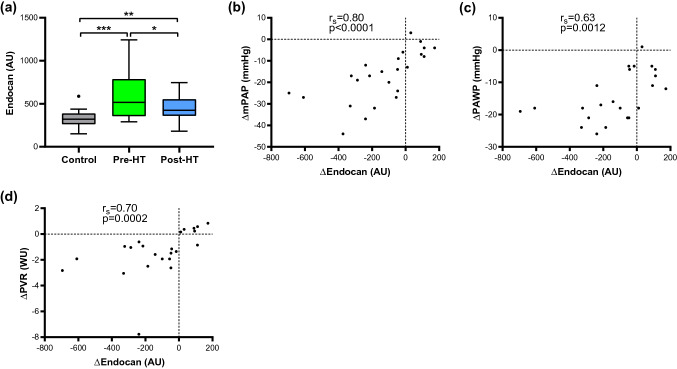
Plasma levels and correlations of endocan with hemodynamic changes following heart transplantation. Level changes (Δ) were calculated using values post-HT–pre-HT and outliers were defined with Tukey’s fence. *HT* heart transplantation, *AU* arbitrary units, *r*_*s*_ Spearman’s correlation coefficient, *mPAP* mean pulmonary arterial pressure, *PAWP* pulmonary arterial wedge pressure, *PVR* pulmonary vascular resistance, *WU* wood units. *p < 0.01; **p < 0.001; ***p < 0.0001

**Fig. 3:**
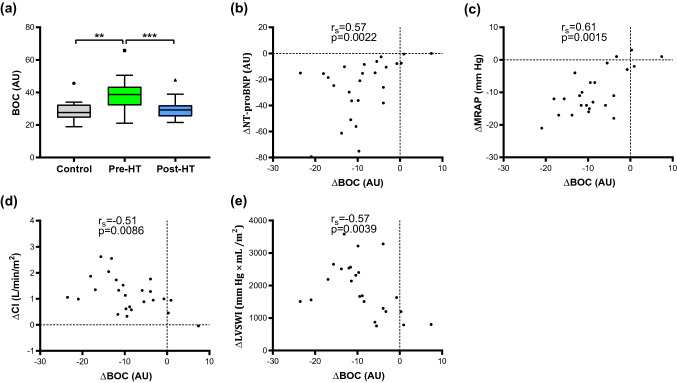
Plasma levels and correlations of BOC with changes in hemodynamics following heart transplantation. Level changes (Δ) were calculated using values post-HT–pre-HT and outliers were defined with Tukey’s fence. *HT* heart transplantation, *AU* arbitrary units, *r*_*s*_ Spearman’s correlation coefficient, *BOC* brother of CDO, *NT-proBNP* N-terminal pro b-type natriuretic peptide, *MRAP* mean right atrial pressure, *CI* cardiac index, *LVSWI* left-ventricular stroke work index. **p < 0.001; ***p < 0.0001

The levels of tumour-related proteins in controls and patients at baseline and after HT are presented in Table 3 and supplementary Table 2 (FDR, Q = 0.01). In end-stage HF patients, plasma levels of endocan (Fig. 2a), BOC (Fig. 3a), CPE and kallikrein 11 were elevated compared to the controls (*p* < 0.01). After HT and reversal of HF and pre-existing PH, these levels decreased vs. pre-HT (*p* < 0.01), towards the controls’ levels, with normalization of BOC and CPE levels.

**Table 3:** Tumour-related proteins’ levels in controls and patients before and 1-year after heart transplantation

Protein (AU)	Control (*n* = 20)		Pre-HT (*n* = 26)		Post-HT (*n* = 26)		Δ (Post-HT–Pre-HT)		*p* value		
	*n*	Median (IQR)	*n*	Median (IQR)	*n*	Median (IQR)	*n*	Median (IQR)	Pre-HT vs. C	Post-HT vs. C	Pre-HT vs. Post-HT
NT-proBNP	20	1.1 (1.1–1.2)	26	24 (11–40)	26	2 (1.4–5.8)	26	− 17 (− 37 to − 8.4)	3.6 × 10^–13^*	5.0 × 10^–5^*	3.0 × 10^–8^*
AP-N	20	21 (19–24)	26	26 (22–38)	26	20 (17–27)	26	− 5.4 (− 16 to − 2.5)	0.0020*	0.45	7.5 × 10^–6^*
BOC	20	28 (24–33)	26	39 (32–44)	26	29 (25–32)	26	− 9.6 (− 13 to − 3.9)	0.00013*	0.61	3.3 × 10^–6^*
CEACAM1	19	132 (124–138)	25	157 (138–174)	26	134 (123–146)	24	− 27 (− 37 to − 5.5)	1.5 × 10^–5^*	0.72	6.6 × 10^–6^*
Cornulin	19	37 (29–57)	25	17 (10–25)	26	24 (15–34)	25	4.8 (2.1 to 11)	4.1 × 10^–7^*	0.00045*	0.0020*
CPA1	20	15 (10–22)	26	25 (18–45)	26	21 (11–30)	26	− 6.5 (− 17 to 1.1)	0.00058*	0.099	0.0051*
CPB1	20	12 (7.7–15)	26	19 (15–33)	26	12 (8–17)	26	− 7.9 (− 16 to − 4.5)	2.4 × 10^–5^*	0.75	5.7 × 10^–7^*
CPE	19	9.8 (7.2–11)	25	12 (9.5–15)	26	8.1 (7.5–9.3)	25	− 3.5 (− 6 to − 1.9)	0.0040*	0.037	1.2 × 10^–7^*
Endocan	19	320 (261–388)	25	516 (355–787)	26	424 (359–554)	25	− 56 (− 263 to 33)	7.7 × 10^–5^*	0.00050*	0.0088*
Furin	19	8.4 (7.4–11)	25	11 (9–14)	26	8.1 (7.3–11)	25	− 2.1 (− 4.3 to − 0.0087)	0.0070*	0.79	0.0020*
Kallikrein 11	19	27 (22–33)	25	55 (43–76)	26	41 (30–55)	25	− 14 (− 24 to − 1.5)	1.4 × 10^–7^*	0.00033*	0.00012*
Kallikrein 14	19	71 (52–79)	25	84 (66–116)	26	67 (55–90)	25	− 16 (− 33 to 0.15)	0.0065*	0.23	0.0013*
Mucin-16	19	15 (12–21)	25	91 (20–238)	26	16 (10–19)	25	− 58 (− 199 to − 4.8)	6.0 × 10^–13^*	0.97	8.2 × 10^–6^*
Vimentin	19	6 (4–9.4)	25	15 (11–21)	26	10 (8.1–14)	25	− 2.5 (− 9.1 to − 0.78)	2.4 × 10^–7^*	0.0014*	0.0018*

*C* control, *HT* heart transplantation, *IQR* interquartile range, *AU* arbitrary units, *NT-proBNP* N-terminal pro b-type natriuretic peptide, *AP-N* aminopeptidase N, *CEACAM1* carcinoembryonic antigen-related cell adhesion molecule 1, *CPA1* carboxypeptidase A1, *CPB1* carboxypeptidase B, *CPE* carboxypeptidase E

*Indicates a difference (*p* < 0.01; FDR, Q = 0.01)

### Plasma ∆endocan, ∆BOC, ∆CPE, and ∆kallikrein 11 correlate with hemodynamic changes following heart transplantation

Correlations of changes (∆) between proteins’ levels with ∆NT-proBNP and ∆hemodynamic parameters following HT are presented in Table 4. ∆endocan correlated with ∆mPAP, ∆PAWP, and PVR (Fig. 2b–d; *p* < 0.01). ∆BOC correlated with ∆NT-proBNP, ∆MRAP, ∆CI, and ∆LVSWI (Fig. 3b–e; *p* < 0.01). ∆CPE correlated with ∆mPAP, ∆PVR and ∆CI (*p* < 0.01). ∆kallikrein 11 correlated with ∆NT-proBNP and ∆MRAP (*p* < 0.01).

**Table 4:** Correlation analysis between changes (Δ) in proteins’ levels with NT-proBNP and Δhemodynamic parameters

ΔVariable	mPAP (mmHg)		PAC (mL/mmHg)		PVR (WU)		PAWP (mmHg)		MRAP (mmHg)		NT-proBNP (AU)		CI (L/min/m^2^)		LVSWI (g/beat/m^2^)	
	*n*	*r* (*p* value)	*n*	*r* (*p* value)	*n*	*r* (*p* value)	*n*	*r* (*p* value)	*n*	*r* (*p* value)	*n*	*r* (*p* value)	*n*	*r* (*p* value)	*n*	*r* (*p* value)
AP-N	25	− 0.19 (0.35)	25	0.15 (0.47)	24	− 0.22 (0.31)	24	− 0.24 (0.25)	24	0.16 (0.46)	26	0.22 (0.28)	25	− 0.33 (0.11)	24	− 0.13 (0.55)
BOC	25	0.37 (0.069)	25	0.015 (0.94)	24	0.27 (0.21)	24	0.41 (0.049)	24	0.61 (0.0015)*	26	0.57 (0.0022)*	25	− 0.51 (0.0086)*	24	− 0.57 (0.0039)*
CEACAM1	24	0.29 (0.17)	24	− 0.13 (0.56)	23	0.3 (0.17)	23	0.16 (0.47)	25	0.088 (0.69)	25	0.12 (0.58)	24	− 0.65 (0.00053)*	23	− 0.15 (0.49)
Cornulin	24	0.17 (0.42)	24	− 0.3 (0.16)	23	0.05 (0.82)	23	0.19 (0.38)	25	− 0.16 (0.46)	25	− 0.35 (0.082)	24	0.072 (0.74)	23	0.22 (0.32)
CPA1	25	0.24 (0.25)	25	0.13 (0.53)	24	− 0.014 (0.95)	24	0.13 (0.54)	24	0.32 (0.13)	26	0.36 (0.071)	25	− 0.19 (0.36)	24	− 0.2 (0.36)
CPB1	25	0.16 (0.45)	25	0.26 (0.21)	24	0.016 (0.94)	24	− 0.094 (0.66)	24	0.22 (0.30)	26	0.2 (0.34)	25	− 0.0023 (0.99)	24	− 0.15 (0.49)
CPE	24	0.5 (0.012)*	24	− 0.046 (0.83)	23	0.52 (0.011)*	23	0.38 (0.074)	23	0.47 (0.024)	25	0.47 (0.019)	24	− 0.59 (0.0022)*	23	− 0.28 (0.19)
Endocan	24	0.8 (2.4 × 10^–6^)*	24	− 0.27 (0.20)	23	0.7 (0.00022)*	23	0.63 (0.0012)*	25	0.37 (0.084)	25	0.47 (0.019)	24	− 0.21 (0.32)	23	− 0.38 (0.072)
Furin	24	0.064 (0.77)	24	− 0.13 (0.55)	23	0.19 (0.38)	23	0.044 (0.84)	23	0.12 (0.58)	25	0.057 (0.79)	24	0.029 (0.89)	23	0.055 (0.80)
Kallikrein 11	24	0.072 (0.74)	24	0.1 (0.64)	23	0.19 (0.38)	23	0.24 (0.26)	25	0.59 (0.0033)*	25	0.66 (0.00034)*	24	− 0.13 (0.54)	23	− 0.19 (0.39)
Kallikrein 14	24	0.22 (0.29)	24	− 0.2 (0.34)	23	0.45 (0.033)	23	0.074 (0.74)	25	− 0.073 (0.74)	25	− 0.043 (0.84)	24	− 0.083 (0.70)	23	0.043 (0.84)
Mucin-16	24	0.063 (0.77)	24	0.17 (0.42)	23	0.014 (0.95)	23	− 0.056 (0.80)	25	0.41 (0.051)	25	0.55 (0.0047)*	24	− 0.25 (0.24)	23	− 0.32 (0.13)
Vimentin	24	0.45 (0.029)	24	− 0.41 (0.04)	23	0.46 (0.027)	23	0.28 (0.19)	25	0.12 (0.60)	25	0.26 (0.20)	24	− 0.33 (0.11)	23	− 0.33 (0.12)

Delta (Δ) was calculated as (Post-HT) − (Pre-HT)

*r*_*s*_ Spearman’s correlation coefficient, *AU* arbitrary units, *WU* wood units, *mPAP* mean pulmonary arterial pressure, *PAC* pulmonary arterial compliance, *PVR* pulmonary vascular resistance, *PAWP* pulmonary arterial wedge pressure, *MRAP* mean right atrial pressure, *NT-proBNP* N-terminal pro b-type natriuretic peptide, *CI* cardiac index, *LVSWI* left-ventricular stroke work index, *AP-N* aminopeptidase N, *CEACAM1* carcinoembryonic antigen-related cell adhesion molecule 1, *CPA1* carboxypeptidase A1, *CPB1* carboxypeptidase B, *CPE* carboxypeptidase E

*Indicates a significant correlation (*p* < 0.013; FDR, Q = 0.1)

### Other tumour-related proteins in end-stage heart failure patients

In end-stage HF patients, plasma levels of AP-N, CEACAM1, CPA1, CPB1, furin, kallikrein 14, mucin-16, and vimentin were elevated compared to controls (*p* < 0.01). After HT and reversal of HF and pre-existing PH, these levels decreased vs. pre-HT (*p* < 0.01), towards the controls’ levels. Conversely, in HF patients, plasma cornulin levels were low compared to controls (*p* < 0.0001), but increased after HT vs. pre-HT (*p* < 0.01), towards the controls’ levels (*p* < 0.001). ∆CEACAM1 correlated with CI, whereas ∆mucin-16 correlated with ∆NT-proBNP (*p* < 0.01). ∆AP-N, ∆CPA1, ∆CPB1, ∆furin, ∆kallikrein 14, ∆vimentin, and ∆cornulin did not correlate with changes in hemodynamics or NT-proBNP (Table 4).

### Plasma endocan in PH-LHD patients following heart transplantation

A subgroup analysis of the plasma levels pre-HT and post-HT between HF without PH (*n* = 7) and PH-LHD patients (*n* = 19) was performed for BOC, CEACAM1, CPE, endocan, kallikrein 11, and mucin-16 (supplementary Table 3). Plasma endocan levels pre-HT were higher in PH-LHD compared to HF without PH group (*p* < 0.001). No differences were found in the other proteins.

### Discussion

Developing a multi-marker panel reflecting different pathophysiological mechanisms underlying HF may be the future approach for individualized phenotyping and management of HF [9], and potentially PH-LHD. In the present study, we found that in end-stage HF patients, elevated endocan, BOC, CPE, and kallikrein 11 levels decreased after HT towards controls’ levels. Moreover, level changes of these proteins correlated with improved hemodynamics after HT. Our results suggest that endocan, BOC, CPE, and kallikrein 11 may reflect different pathophysiologic mechanisms and be potential biomarkers in HF and PH-LHD.

Endocan is a dermatan sulfate proteoglycan expressed by vascular endothelial cells, cardiomyocytes, and pulmonary capillaries. By virtue of its ability to interact with bioactive proteins, endocan regulates a wide range of biological processes including proliferation, neovascularization, and cellular adhesion [26]. Endocan has been implicated in vascular diseases, endothelium-dependent pathologies, and inflammatory processes including sepsis [27] and systemic sclerosis [28]. Also, endocan has been proposed as an indicator of endothelium activation [27] and dysfunction in septic patients [29]. Elevated circulating endocan levels has been reported in various conditions including hypertension and atherosclerosis [30] as well as in malignant lymphoma, renal cell carcinoma [26], and lung cancer [31].

**Fig. 4:**
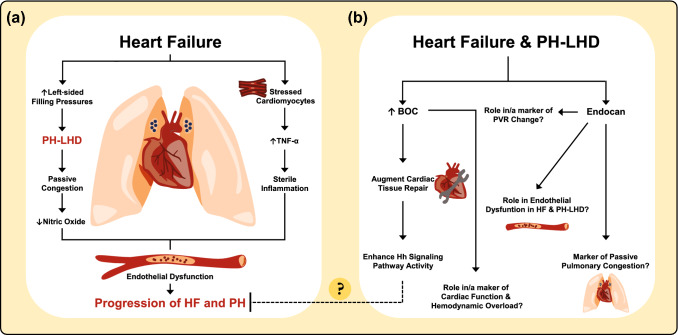
Endothelial dysfunction in the progression of heart failure and pulmonary hypertension; and possible roles of plasma endocan and BOC. **a** Mechanisms involved in the progression of heart failure (HF) and pulmonary hypertension due to left heart disease (PH-LHD). In HF, along with cardiomyocyte hypertrophy and apoptosis, endothelial dysfunction leads to reduced myocardial perfusion and progression of HF. In PH-LHD, endothelial dysfunction may trigger excessive vasoconstriction and vascular remodeling. **b** Hypothetical mechanism of elevated plasma brother of CDO (BOC) in response to HF, i.e., whether activation of the Hedgehog (Hh) signaling augments the progression of HF, as well as possible (patho-)physiological and clinical roles of both BOC and endocan. *PVR* pulmonary vascular resistance

Secondary to HF, malfunctioning and hemodynamically stressed cardiomyocytes result in cytokine release of tumour necrosis factor-α (TNF-α) and interleukin-1β, eliciting a sterile inflammation in the heart. As a result, cardiomyocyte apoptosis and hypertrophy, myofibroblast differentiation, and endothelial dysfunction ensue, leading to reduced myocardial perfusion, ventricular remodeling and subsequent progression of HF [32, 33]. Endothelial dysfunction, defined as an imbalance in the production of vasoactive substances, i.e., increased endothelin-1 expression and reduced nitric-oxide availability, plays a central role in the pathophysiology of HF [33], PH-LHD [34], and PAH [8] (Fig. 4a). Apart from being linked to cardiovascular risk factors, endothelial dysfunction predicts adverse clinical events, and its grade is analogous to the functional capacity and the severity of HF [33]. A recent study of chronic HF patients showed that apart from plasma endocan being elevated compared with healthy controls and patients with coronary artery disease, it emerged as an independent prognosticator of HF-related hospitalization and mortality [35]. In a rat model of PAH, endocan levels were elevated in the serum and lungs, and knockdown of endocan reversed monocrotaline-induced pulmonary vascular remodeling and reduced right-ventricular pressure. A subsequent in vitro experiment on rat pulmonary microvascular endothelial cells displayed the important interplay between TNF-α and endocan, as TNF-α upregulation induced endocan expression, whereas endocan inhibition prevented TNF-α-induced vascular permeability [36]. In the present study, we found that endocan levels were elevated in end-stage HF patients with or without PH-LHD compared to controls. After HT and reversal of HF and concomitant PH, endocan levels decreased towards controls’ levels. The following subgroup analysis revealed that plasma endocan is higher in patients with HF and PH-LHD compared to HF patients without PH, potentially suggesting endocan to be more PH-specific. Moreover, Δendocan correlated with ΔmPAP, ΔPAWP, and ΔPVR, reflecting the state of PH, passive pulmonary congestion as well as pulmonary vascular tone following HT, respectively. Taken together, elevated plasma endocan, may, theoretically have a role in endothelial dysfunction in HF patients with PH, as reflected by the correlation with PVR (Fig. 4b). It is also possible that endocan may be linked to, or involved in pulmonary vasoconstriction and pulmonary vascular remodeling, as endothelial dysfunction is a well-known trigger of these processes in PH-LHD [3]. Thus, it is encouraging to investigate the role of endocan, its interactions with TNF-α as well as its clinical implications as a biomarker of pulmonary congestion and potentially endothelial dysfunction in HF and PH-LHD.

The hedgehog (Hh) signaling pathway is crucial in embryogenesis, organ development, as well as in adult tissue repair and homeostasis [37, 38]. Aberrant Hh signaling has emerged as an important pathway in human cancer, including basal cell carcinoma and medulloblastoma. BOC is a transmembrane co-receptor which through unknown molecular mechanisms enhances Hh pathway activity and facilitates Hh ligand binding to its receptor, Patched 1, which elicits responses in a dose-dependent manner [38]. In an adult murine model, Hh signaling was shown to be critical in the maintenance of coronary arteries, and ablation of Hh signaling resulted in coronary vasculature dropout, cardiomyocyte apoptosis, ventricular failure, and death. In an ensuing experiment, reduction of endogenous Hh signaling after myocardial infarction aggravated heart function and increased infarction size [39]. In another adult murine model, intramyocardial gene therapy with Shh—a Hh-specific ligand—after acute and chronic myocardial ischemia, resulted in preserved left-ventricular function by augmenting neovascularisation, as well as reducing fibrosis and cardiomyocyte apoptosis [40]. In the present study, plasma BOC levels were elevated in HF patients irrespective of PH compared to controls. These levels decreased and normalized upon the reversal of HF and PH after HT. The decrease of BOC following HT correlated with decreased NT-proBNP and MRAP as well as increased CI and LVSWI, reflecting decreased cardiac overload and improved cardiac function after HT. All in all, whether plasma BOC elevation is an endogenous response to counteract HF progression through enhancing Hh signaling reception and thereby augmenting cardiac tissue repair in chronic HF remains to be investigated (Fig. 4a, b).

CPE or enkephalin convertase is a member of metallocarboxypeptidase gene family and is most abundantly found in endocrine tissues, but also in heart and lungs [41]. CPE is involved in the biosynthesis of numerous prohormones and neurotransmitters. CPE is overly expressed in a variety of cancers including neuroendocrine tumours and small-cell lung carcinoma, and its abundance therein promotes neuropeptide biosynthesis, resulting in autocrine tumour growth [42]. In the fields of cardiovascular physiology and pathobiology, it has been proposed that CPE may be involved in atrial natriuretic peptide synthesis in rat hearts [43]. Moreover, a series of studies of Chinese patients found that specific CPE gene polymorphisms may be linked to increased severity of coronary atherosclerosis [44–46]. In the present study, plasma CPE levels were elevated before HT compared to controls, which thereafter decreased after HT towards controls’ levels. Plasma-level changes in CPE correlated with changes in mPAP, PVR, and CI. Whether these associations infer causality between CPE, HF, and pulmonary vascular disease remains to be further elaborated.

Kallikrein 11 is a member of soluble serine proteases and is regulated in a steroid hormone-dependent manner. It is highly expressed in human prostatic and tracheal tissues, but also present in lungs and serum [47]. Despite several studies addressing its potential diagnostic or prognostic properties in prostate, ovarian [47], and lung cancer [48], the precise physiological function of kallikrein 11 remains largely unknown. In assessing the enzymatic function and its physiological substrates, unlike some kallikreins, kallikrein 11 is incapable of cleaving kininogen [49], an activator of the kallikrein–kinin system that has been implicated in left-ventricular dysfunction [50]. Instead, it cleaves and degrades insulin-like growth factor-binding protein 3 (IGFBP3) [51]. As a carrier protein, IGFBP3 extends the half-life of IGFs, and upon the cleavage of the IGF–IGFBP3 complex, IGFs are released to bind and activate IGF-1 receptor signaling [52]. In dilated cardiomyopathy, elevated IGFBP3 and IGF-1 mRNA tissue expressions have been reported in comparison to controls [53]. Moreover, IGF-1 signaling has been implicated in cardiac ageing and dysfunction [54]. Another study reported that elevated plasma IGF-1 in HF patients appeared to be associated with angiotensin-converting enzyme inhibitor (ACEi) treatment and increased risk of cardiovascular mortality [55]. Herein, kallikrein 11 plasma levels were elevated in advanced HF patients in comparison to controls. These levels decreased after HT matching controls’ levels. Furthermore, a decrease in plasma kallikrein 11 correlated with a decrease in NT-proBNP and MRAP, reflecting an alleviated cardiac hemodynamic overload. Hypothetically, elevated circulating kallikrein 11 in HF may have a role in promoting cardiac ageing and accelerating ventricular dysfunction through increasing the bioactivity of IGF-1. Hence, the role of plasma kallikrein 11 in HF warrants further investigation.

Moreover, plasma CEACAM1 and mucin-16 levels were elevated in HF patients, with these levels decreasing after HT towards controls’ levels. The decrease of CEACAM1 and mucin-16 correlated with improved CI and NT-proBNP, respectively, supporting the previously reported association between mucin-16 and volume overload [18]. Intriguingly, a study showed that CEACAM1 upregulation after hypoxic cardiomyocyte injury promoted unfavorable cardiac remodeling by inducing apoptosis [56]. Whether elevated CEACAM1 levels play a role in the chronic progression of HF remains to be investigated.

### Strengths and limitations

Although concordant with the size of other studies, the relatively small population and the lack of validation cohorts constitute limitations. Despite the inability to provide absolute protein concentrations, tissue-specific expression and differentiation of protein isoforms, PEA, compared to conventional multiplex immunoassays, warrant high specificity and sensitivity [22], which is crucial in the process of identifying biomarker candidates for future clinical utility. Thus, the use of PEA and the invasive hemodynamic measurements constitutes major strengths in our study. Noteworthy is, however, that the present study is hypothesis generating and our results do not necessarily imply causality. Hence, our results do not allow for definite mechanistical conclusions. Factors including comorbidities, age and sex disparities, diurnal variations, and medication intake may have affected the proteins’ levels. Although it is well established that β-blockers improve left-ventricular function [57] and ACEi increase CO and attenuate left-ventricular wall stress [58], their postoperative withdrawal effects remain unknown. Analogously, antihypertensive and HF-specific medications including ACEi, angiotensin II receptor blockers, and calcium channel antagonists attenuate vascular inflammation and/or endothelial dysfunction [59], specifically valsartan and amlodipine, which may affect plasma endocan levels [27]. Conversely, first- and second-generation β-blockers and diuretics have not been shown to affect inflammation. Moreover, the role of diuretics in endothelial function remains unknown [59]. Although the effects of immunosuppressants on plasma protein levels have not been investigated in the present study, calcineurin and mTOR inhibitors are associated with endothelial dysfunction and increased risk of cardiovascular morbidity [60], whereas mycophenolate mofetil reduces immune-mediated vascular injury and possibly exert positive effects on endothelial function [61]. Furthermore, given the large number of statistical tests conducted, false-positive results may be present, even though FDR was used to accommodate for mass significance. Larger studies are necessary to confirm and validate our findings.

### Conclusions

In the present study, we identified the tumour-related proteins endocan, BOC, kallikrein 11, CPE, CEACAM1, and mucin-16 in end-stage HF patients before and after HT. Specifically, the decrease of high plasma levels of endocan in HF patients after HT was associated with improved mPAP, PAWP, and PVR. Moreover, the decrease after HT of elevated BOC levels was associated with decreased MRAP and NT-proBNP, as well as increased CI and LVSWI. Our results suggest that endocan may be a potential biomarker reflecting the state of PH, pulmonary congestion, and potentially endothelial dysfunction in HF and PH-LHD. Additionally, plasma BOC may be a biomarker candidate, potentially reflecting the hemodynamic overload and heart function in HF, irrespective of concomitant PH. The exact roles of endocan and BOC as well as their potential clinical applicability in HF and PH-LHD remain to be elaborated in future studies.

## Electronic supplementary material

Below is the link to the electronic supplementary material.

Supplementary file1 (DOCX 41 kb)
