# Supplementary material for: Elevated plasma endocan and BOC in heart failure patients decrease after heart transplantation in association with improved hemodynamics
Source: Heart Vessels. 2020 Jul 10;35(11):1614–28. doi: 10.1007/s00380-020-01656-3 (PMC7502449; doi:10.1007/s00380-020-01656-3)
Supplement: Supplementary file 1 — Supplementary file1 (DOCX 41 kb) [file 380_2020_1656_MOESM1_ESM.docx]

**Journal name: Heart and Vessels**

**Title: Elevated Plasma Endocan and BOC in Heart Failure Patients Decrease After Heart Transplantation in Association with Improved Hemodynamics**

**Authors:** Salaheldin Ahmed PhD student ^a,b^, Abdulla Ahmed PhD student ^a,b^, Habib Bouzina, PhD student ^a,b^, Jakob Lundgren MD, PhD ^a,b^ and Göran Rådegran, Associate Prof, DMSc, MD, MS Eng Phys ^a,b^

**Affiliations:** ^a^Department of Clinical Sciences Lund, Cardiology, Lund University. ^b^ The Hemodynamic Lab, The section for Heart Failure and Valvular Disease, VO. Heart and Lung Medicine, Skåne University Hospital, Lund, Sweden.

**Corresponding author:** Salaheldin Ahmed

E-Mail: [salaheldin.ahmed@med.lu.se](mailto:salaheldin.ahmed@med.lu.se)

Address: EA15, Skåne University Hospital, 22185 Lund, Sweden

Fax number: 0046 - (0)46 – 307984

**Supplementary Table 1. Additional hemodynamic parameters of heart failure patients pre and one-year post heart transplantation**

| **Hemodynamic parameter** | **Pre-HT (n=7)** | | **Post-HT (n=7)** | | **Pre-HT (n=19)** | | **Post-HT (n=19)** | | **Pre-HT (n=26)** | | **Post-HT (n=26)** | | **P-value** |
| --- | --- | --- | --- | --- | --- | --- | --- | --- | --- | --- | --- | --- | --- |
|  | **n** | **Median (IQR)** | **n** | **Median (IQR)** | **n** | **Median (IQR)** | **n** | **Median (IQR)** | **n** | **Median (IQR)** | **n** | **Median (IQR)** | **Post-HT vs. Pre-HT** |
| TPG (mmHg) | 6 | 4 (3,3 ‒ 6) | 7 | 8 (3 ‒ 10) | 18 | 10 (7 ‒ 14) | 19 | 8 (5 ‒ 10) | 24 | 8.5 (6 ‒ 12) | 26 | 8 (5 ‒ 10) | 0.17 |
| DPG (mmHg) | 6 | 1 (-0,25 ‒ 1,8) | 7 | 2 (0 ‒ 3) | 18 | 2 (0 ‒ 4.3) | 19 | 2 (-1 ‒ 4) | 24 | 1 (0 ‒ 3.8) | 26 | 2 (-0.25 ‒ 4) | 0.8 |
| Heart rate (beats/min) | 6 | 76 (60 ‒ 85) | 7 | 89 (71 ‒ 90) | 19 | 73 (69 ‒ 78) | 19 | 81 (73 ‒ 87) | 25 | 73 (69 ‒ 82) | 26 | 82 (73 ‒ 89) | 0.063 |
| SV (mL/beat) | 6 | 55 (47 ‒ 56) | 7 | 73 (65 ‒ 81) | 19 | 45 (34 ‒ 59) | 19 | 72 (66 ‒ 78) | 25 | 48 (35 ‒ 58) | 26 | 72 (66 ‒ 78) | 4.2×10^-7^***** |
| PVR index (WU/m^2^) | 6 | 2,5 (1,7 ‒ 2,9) | 7 | 3,2 (0,81 ‒ 3,7) | 18 | 6.3 (4.7 ‒ 7.2) | 19 | 2.7 (1.8 ‒ 3.7) | 24 | 5.1 (2.9 ‒ 6.9) | 26 | 2.8 (1.7 ‒ 3.7) | 5.3×10^-5^***** |
| RVSWI (mmHg×mL/m^2^) | 6 | 178 (26 ‒ 414) | 7 | 335 (292 ‒ 508) | 18 | 483 (296 ‒ 732) | 18 | 411 (306 ‒ 520) | 25 | 362 (294 ‒ 615) | 25 | 429 (317 ‒ 516) | 0.64 |
| SaO_2_ (%) | 6 | 96 (95 ‒ 97) | 6 | 97 (93 ‒ 98) | 19 | 96 (93 ‒ 96) | 17 | 97 (96 ‒ 98) | 25 | 96 (94 ‒ 97) | 23 | 97 (96 ‒ 98) | 0.046 |
| SvO_2_ (%) | 6 | 59 (49 ‒ 70) | 7 | 69 (62 ‒ 73) | 19 | 49 (46 ‒ 57) | 19 | 70 (67 ‒ 72) | 25 | 52 (47 ‒ 60) | 26 | 69 (66 ‒ 72) | 8.3×10^-7^***** |
| a-vO_2_ diff (mL O_2_/L) | 6 | 64 (49 ‒ 88) | 6 | 42 (38 ‒ 50) | 19 | 74 (69 ‒ 82) | 17 | 42 (40 ‒ 51) | 25 | 74 (63 ‒ 81) | 23 | 42 (40 ‒ 51) | 2.4×10^-6^***** |

(*****) p<0.0003. FDR<0.01. Subgroups: (n=7), heart failure patients without pulmonary hypertension; (n=19), patients with pulmonary hypertension due to left heart disease. (n=26), the entire study population pooled.

**Abbreviations:** IQR, interquartile range; WU, wood units; TPG, transpulmonary pressure gradient; DPG, diastolic pressure gradient; SV, stroke volume; SVI, stroke volume index; PVR, pulmonary vascular resistance; RVSWI, right ventricular stroke work index; SaO_2_, arterial oxygen saturation; SvO_2_, mixed venous oxygen saturation and a-vO_2_ diff, arteriovenous oxygen difference.

**Supplementary Table 2. Tumour related proteins levels’ in controls and patients before and one-year after heart transplantation.**

| **Protein (AU)** | Control (n=20) | | Pre-HT (n=26) | | Post-HT (n=26) | | Δ (Post-HT–Pre-HT) | | P-value | | |
| --- | --- | --- | --- | --- | --- | --- | --- | --- | --- | --- | --- |
|  | n | Median (IQR) | n | Median (IQR) | n | Median (IQR) | n | Median (IQR) | Pre-HT vs C | Post-HT vs C | Pre- vs Post-HT |
| 5'-nucleotidase | 19 | 655 (535 ‒ 716) | 25 | 1636 (853 ‒ 2194) | 26 | 978 (731 ‒ 1884) | 25 | -197 (-682 ‒ 229) | 9.8×10^-7^* | 0.00041* | 0.063 |
| AMBP | 20 | 96 (88 ‒ 108) | 26 | 107 (98 ‒ 119) | 26 | 131 (115 ‒ 139) | 26 | 20 (11 ‒ 34) | 0.023 | 3.4×10^-8^* | 1.3×10^-6^* |
| BLM-H | 20 | 32 (26 ‒ 37) | 26 | 31 (27 ‒ 42) | 26 | 34 (28 ‒ 44) | 26 | 4.8 (-11 ‒ 12) | 0.82 | 0.36 | 0.53 |
| CA9 | 19 | 5.1 (3.4 ‒ 6.2) | 25 | 10 (6.1 ‒ 17) | 26 | 7.9 (5.5 ‒ 11) | 25 | -1.1 (-5 ‒ 1.6) | 0.00011* | 0.0012* | 0.22 |
| Cathepsin Z | 20 | 19 (16 ‒ 22) | 26 | 20 (15 ‒ 24) | 26 | 25 (20 ‒ 35) | 26 | 5.2 (0.84 ‒ 12) | 0.58 | 0.0034* | 0.00047* |
| CDKN1A | 19 | 25 (9.2 ‒ 36) | 25 | 12 (6.6 ‒ 25) | 26 | 17 (9.4 ‒ 28) | 25 | 2.3 (-4.8 ‒ 15) | 0.023 | 0.24 | 0.16 |
| CEACAM5 | 19 | 3.4 (2.5 ‒ 6.9) | 25 | 4.3 (3 ‒ 9.5) | 26 | 3.7 (2.5 ‒ 5.5) | 25 | -1 (-2.6 ‒ 0.17) | 0.30 | 0.99 | 0.0087* |
| Contactin-1 | 20 | 6 (5 ‒ 6.7) | 26 | 4.9 (4.3 ‒ 6.2) | 26 | 3.9 (3.5 ‒ 4.9) | 26 | -0.79 (-1.7 ‒ -0.52) | 0.047 | 9.1×10^-7^* | 1.6×10^-5^* |
| Cystatin B | 20 | 14 (10 ‒ 20) | 26 | 29 (19 ‒ 53) | 26 | 20 (18 ‒ 35) | 26 | -5.5 (-31 ‒ 1.5) | 8.8×10^-6^* | 0.0012* | 0.018 |
| EpCAM | 20 | 9.1 (7.6 ‒ 29) | 26 | 18 (7.8 ‒ 29) | 26 | 12 (8.7 ‒ 21) | 26 | -1.3 (-12 ‒ 5.1) | 0.67 | 0.73 | 0.22 |
| Gastrotropin | 20 | 2.2 (1.9 ‒ 2.8) | 26 | 3.6 (2.8 ‒ 5.5) | 26 | 4.4 (3.6 ‒ 7.6) | 26 | 0.72 (-0.41 ‒ 2.8) | 0.00076* | 1.3×10^-5^* | 0.020 |
| Glyoxalase I | 20 | 84 (55 ‒ 179) | 26 | 231 (174 ‒ 320) | 26 | 216 (161 ‒ 302) | 26 | -32 (-105 ‒ 54) | 4.3×10^-5^* | 0.00024* | 0.39 |
| Kallikrein 13 | 19 | 22 (17 ‒ 31) | 25 | 32 (23 ‒ 39) | 26 | 29 (24 ‒ 37) | 25 | -2.2 (-9 ‒ 4.7) | 0.0095* | 0.035 | 0.23 |
| Kallikrein 6 | 20 | 6.5 (5.8 ‒ 7.7) | 26 | 8.9 (7.7 ‒ 11) | 26 | 10 (8.5 ‒ 12) | 26 | 0.95 (-0.37 ‒ 2.2) | 3.4×10^-5^* | 1.7×10^-6^* | 0.033 |
| Kallikrien 8 | 19 | 53 (42 ‒ 62) | 25 | 61 (52 ‒ 69) | 26 | 63 (57 ‒ 76) | 25 | 6.7 (-6 ‒ 16) | 0.054 | 0.0061* | 0.11 |
| LYPD3 | 19 | 14 (11 ‒ 18) | 25 | 11 (9.6 ‒ 13) | 26 | 11 (8.7 ‒ 12) | 25 | -0.72 (-2.5 ‒ 0.22) | 0.0095* | 0.00055* | 0.11 |
| Mesothelin | 19 | 2.9 (2.2 ‒ 4.7) | 25 | 4.3 (3.5 ‒ 5.9) | 26 | 3.3 (2.5 ‒ 4.7) | 25 | -0.7 (-1.9 ‒ 0.29) | 0.014 | 0.46 | 0.012 |
| MetAP2 | 19 | 31 (17 ‒ 38) | 25 | 24 (19 ‒ 32) | 26 | 27 (20 ‒ 36) | 25 | 2.2 (-12 ‒ 15) | 0.78 | 0.92 | 0.87 |
| MIA | 19 | 796 (723 ‒ 896) | 25 | 823 (736 ‒ 879) | 26 | 853 (762 ‒ 991) | 25 | 72 (-3.6 ‒ 163) | 0.73 | 0.091 | 0.016 |
| Midkine | 19 | 72 (51 ‒ 85) | 25 | 100 (79 ‒ 137) | 26 | 103 (66 ‒ 146) | 25 | 2.6 (-39 ‒ 38) | 0.00027* | 0.0045* | 0.99 |
| Podocalyxin | 19 | 9.6 (9.2 ‒ 11) | 25 | 9.3 (8.6 ‒ 9.9) | 26 | 9.6 (8.8 ‒ 10) | 25 | 0.44 (-0.36 ‒ 1.2) | 0.12 | 0.31 | 0.048 |
| Prostasin | 20 | 289 (239 ‒ 337) | 26 | 449 (344 ‒ 510) | 26 | 402 (340 ‒ 511) | 26 | 6.9 (-52 ‒ 74) | 1.1×10^-5^* | 8.8×10^-6^* | 0.82 |
| Nectin-4 | 19 | 36 (33 ‒ 41) | 25 | 44 (34 ‒ 59) | 26 | 66 (40 ‒ 78) | 25 | 11 (2.7 ‒ 27) | 0.028 | 3.7×10^-6^* | 0.00016* |
| S100A11 | 19 | 4.8 (4.5 ‒ 5.2) | 25 | 5.8 (5.3 ‒ 6.6) | 26 | 6.9 (5.3 ‒ 8.2) | 25 | 0.64 (-1.1 ‒ 3.1) | 0.00050* | 6.5×10^-6^* | 0.090 |
| S100A4 | 19 | 3.7 (2.8 ‒ 4.4) | 25 | 3.1 (2.9 ‒ 3.6) | 26 | 3 (2.6 ‒ 3.4) | 25 | -0.3 (-0.65 ‒ 0.46) | 0.19 | 0.035 | 0.28 |
| SCAMP3 | 19 | 46 (13 ‒ 61) | 25 | 22 (13 ‒ 45) | 26 | 28 (14 ‒ 46) | 25 | 0.042 (-18 ‒ 12) | 0.30 | 0.28 | 0.96 |
| SCGB3A2 | 19 | 3.7 (3 ‒ 4.5) | 26 | 5.6 (3.7 ‒ 8.7) | 26 | 5.9 (4.4 ‒ 8.6) | 26 | 0.7 (-0.48 ‒ 1.8) | 0.013 | 0.0021* | 0.23 |
| SHPS-1 | 20 | 7.9 (7.1 ‒ 10) | 26 | 10 (8.2 ‒ 14) | 26 | 9.2 (7.1 ‒ 12) | 26 | -1.1 (-3.7 ‒ 0.13) | 0.028 | 0.25 | 0.0029* |
| Sortilin | 20 | 54 (48 ‒ 64) | 26 | 63 (61 ‒ 73) | 26 | 59 (52 ‒ 68) | 26 | -7.4 (-14 ‒ 4.8) | 0.0058* | 0.23 | 0.094 |
| TCL1A | 19 | 53 (31 ‒ 92) | 25 | 28 (13 ‒ 45) | 26 | 8 (4.2 ‒ 14) | 25 | -13 (-22 ‒ -6.9) | 0.0014* | 3.7×10^-9^* | 6.0×10^-7^* |
| TFF3 | 20 | 21 (19 ‒ 26) | 26 | 38 (30 ‒ 58) | 26 | 38 (30 ‒ 61) | 26 | -0.82 (-10 ‒ 5.2) | 1.5×10^-7^* | 3.4×10^-5^* | 0.41 |
| TGM2 | 20 | 247 (134 ‒ 325) | 26 | 190 (149 ‒ 252) | 26 | 158 (116 ‒ 214) | 26 | -30 (-85 ‒ 34) | 0.16 | 0.035 | 0.094 |
| WFDC2 | 19 | 69 (59 ‒ 77) | 25 | 150 (111 ‒ 191) | 26 | 145 (101 ‒ 188) | 25 | -4.3 (-52 ‒ 26) | 2.7×10^-11^* | 3.1×10^-10^* | 0.33 |
| VSIG2 | 20 | 7.3 (6.8 ‒ 8.4) | 26 | 15 (10 ‒ 19) | 26 | 16 (14 ‒ 28) | 26 | 3.5 (-1.2 ‒ 7.5) | 1.9e×10^-8^* | 7.4×10^-10^* | 0.038 |
| XPNPEP2 | 19 | 89 (49 ‒ 126) | 25 | 110 (54 ‒ 136) | 26 | 95 (42 ‒ 130) | 25 | -10 (-21 ‒ 2.1) | 0.29 | 0.72 | 0.0018* |

(*) Indicates a difference (p<0.01. FDR<0.01).

Abbreviations: C, Control; HT, heart transplantation; IQR, inter quartile range; AU, arbitrary units; AMBP, protein AMBP; BLM-H, bleomycin hydrolase; CA9, carbonic anhydrase 9; CDKN1A, cyclin-dependent kinase inhibitor 1A or p21; CEACAM5, carcinoembryonic antigen-related cell adhesion molecule 5; Ep-CAM, epithelial cell adhesion molecule; glyoxalase I or lactoylglutathione lyase; LYPD3, Ly6/PLAUR domain-containing protein 3 or C4.4A; MetAP2, methionine aminopeptidase 2; MIA, melanoma-derived growth regulatory protein or melanoma inhibitory activity; SCAMP3, secretory carrier-associated membrane protein 3; SCGB3A2, secretoglobin family 3A member 2; SHPS-1, tyrosine-protein phosphatase non-receptor type substrate 1; TCL1A, T-cell leukemia/lymphoma protein 1A; TFF3, trefoil factor 3; TGM2, protein-glutamine gamma-glutamyltransferase 2; WFDC2, WAP four-disulfide core domain protein 2; V-set and immunoglobulin domain-containing protein 2 (VSIG2) and Xaa-Pro aminopeptidase 2 (XPNPEP2).

**Supplementary table 3. Subgroup analysis of proteins between heart failure and PH-LHD patients**

| **Protein (AU)** | **Pre-HT (n=7)** | **Pre-HT (n=19)** | **P-value** | **Post-HT (n=7)** | **Post-HT (n=19)** | **P-value** |
| --- | --- | --- | --- | --- | --- | --- |
| BOC | 39 (27 ‒ 42) | 37 (32 ‒ 47) | 0.69 | 30 (26 ‒ 33) | 29 (24 ‒ 32) | 0.5 |
| CEACAM1 | 151 (132 ‒ 159) | 160 (138 ‒ 176) | 0.3 | 136 (120 ‒ 157) | 134 (123 ‒ 138) | 0.82 |
| CPE | 9.6 (9.5 ‒ 12) | 13 (11 ‒ 15) | 0.2 | 8.4 (7.9 ‒ 10) | 7.8 (6.8 ‒ 9.2) | 0.15 |
| Endocan | 334 (290 ‒ 393) | 710 (473 ‒ 812) | **0.0008*** | 379 (325 ‒ 504) | 426 (387 ‒ 566) | 0.5 |
| Kallikrein 11 | 46 (38 ‒ 94) | 55 (45 ‒ 76) | 0.84 | 54 (41 ‒ 57) | 38 (30 ‒ 54) | 0.21 |
| Mucin 16 | 116 (16 ‒ 265) | 81 (23 ‒ 226) | 0.93 | 15 (11 ‒ 19) | 17 (9 ‒ 20) | 0.99 |

(*) significant p-values. Data is presented as median (IQR). Subgroups: (n=7), heart failure patients without pulmonary hypertension; (n=19), patients with pulmonary hypertension due to left heart disease (PH-LHD).

Abbreviations: AU, arbitrary units; CEACAM1, carcinoembryonic antigen-related cell adhesion molecule 1; CPE, Carboxypeptidase E and BOC, Brother of CDO.
